# “Timely help” or “one disaster after another”: The impact of potential and transition interim CEO succession on corporate performance

**DOI:** 10.1371/journal.pone.0311281

**Published:** 2024-10-14

**Authors:** Conghui Zhao, Xin Guan, Xinyue Wu

**Affiliations:** 1 School of Management, Henan University of Chinese Medicine, Zhengzhou, Henan, China; 2 College of Business Administration, Capital University of Economics and Business, Beijing, China; Xi’an Jiaotong University City College, CHINA

## Abstract

The succession mode of interim CEO is highly uncertain. The smooth transition function of interim CEO is particularly important in the process of enterprise reform. We divide interim CEOs into potential successors and transition successors. Then we empirically analyze the heterogeneous impact and mechanism of interim CEOs on firm performance. The results show that compared with transition CEO, potential CEO has less negative impact on corporate performance. Interim CEO has a negative impact on enterprise performance by reducing agency efficiency. The mechanism analysis of this paper are as follows. For the potential CEO, strong incentive constraints can effectively mitigate the negative impact of the succession on corporate performance. For a transitional CEO, superior personal capabilities coupled with an effective oversight mechanism represent more optimal solutions. In today’s corporate landscape, the frequencies of executive changes and interim successions are on the rise annually. Our research is tailored to aid enterprises in navigating these transitions seamlessly and in expediting their recovery processes.

## Introduction

The abrupt departure of CEOs during their tenure has become increasingly common among public companies. Concurrently, as executive tenures gradually shorten, instances of interim CEO succession have been on the rise. At the practical level, a significant number of firms in China lack formal succession plans [1], and the professional manager market lags behind that of the West. Interim succession serves not only as a crucial strategy for seamless transition during uncertain crises but also as a means for companies to assess potential successors. As companies confront succession dilemmas, the model of interim CEO succession is becoming more prevalent and adopted. Firms are thus urged to develop and implement formal, long-term applicable succession plans. Academically, CEO succession is a pivotal strategic decision impacting enterprise stability and development, a topic extensively discussed by scholars globally. CEO succession can be broadly categorized into three types. First, ‘relay succession,’ where candidates are evaluated and ascend to the CEO role [2]. Second, ‘competitive succession,’ in which primarily internal candidates compete, with the victor officially succeeding. And third, ‘interim succession,’ applicable in scenarios of sudden CEO departure or when trial assessment of a formal successor is necessitated [3]. Interim CEOs typically have shorter tenures but do not preclude the possibility of a permanent turnaround. In the face of succession crises, numerous organizations employ the services of interim CEOs [4]. Furthermore, demographic similarity influences the appointment of interim CEOs; Davidson et al. (2006) discovered that boards prefer to appoint individuals of similar age as interim CEOs [5].

### Patterns and reasons for interim CEO succession

Interim CEOs fulfill six distinct roles within an organization: seat warmer, contender, groomer, marketer, fixer, and cleaner. The varying succession roles and purposes have a differential impact on firm performance [6]. It has been argued that interim CEOs, as one of the executive turnover methods, also need to consider the reasons for the departure of the former CEO [7]. Zhang (2018) discovered that when former executives must leave, outside directors prefer the external succession model, leading to a usually positive stock impact [8]. In addition, when the executive team fires the incumbent CEO for inefficiency and poor performance, the interim CEO is often succeeded by a salaried “regular” [1]. Such CEOs can be recruited internally or externally [9].

### Economic consequences of interim CEO succession

Interim succession is characterized by its short-term contractual nature, and many scholars maintain reservations about the efficacy of this method as a turnover strategy. A growing number of scholars have investigated the economic implications of interim CEO transitions, exploring their influence on several key areas: firm performance [10], Research and Development (R&D) expenditures [11], long-term investments [12], investment efficiency [13], and innovation investments [14], to name a few. There is no consensus on the economic consequences of interim succession, which are categorized as “adding insult to injury” and “sending carbon to injury”.

Research on negative schools. First, corporate executive change and the accompanying succession of an interim CEO exacerbate the uncertainty of disruptive turnover events for the firm [10]. This is mainly manifested in information asymmetry problems. These problems arise from the interim successor’s lack of authority and legitimacy during his or her tenure [15]. Additionally, there’s stagnation of strategic change in the firm [10] and negative stakeholder attribution [21]. Secondly, the appointment of interim successors can lead to brain drain and internal conflicts within the executive team, resulting in a loss of the firm’s human capital [16]. This is further complicated by the short-term contractual nature of interim succession. Interim CEOs may engage in surplus management, aiming for a smooth promotion [1]. However, this can result in negative feedback from the external market [17].

Research on positive schools. The school argues that firing a CEO for performance reasons can lead to a “revolving door” situation. This is where the board hires and fires multiple CEOs within a short time [16]. In such cases, the costs associated with trial-and-error executive changes significantly exceed the succession costs of an interim CEO. After firing the former CEO, the firm aims to appoint an “unplanned” successor. This is to prevent the internal successor from copying the former CEO’s methods or being influenced by their human capital. Interim CEO succession not only provides firms with additional time to identify a suitable successor [4], but it also minimizes the expenses related to disruptive turnover events. As an emerging succession type, interim succession broadens the opportunities for firms to evaluate and confirm the competencies of their executives.

In conclusion, compared to relay and competitive succession, interim succession, a relatively new type of succession, has been less studied. Research on interim succession indicates that it is a method suitable for crisis situations [4, 18], significantly impacting business operations and performance [9, 10, 19]. The occurrence of interim CEO succession has been increasing annually in tandem with the rising rate of executive turnover. However, few scholars have explored the motivations and roles of interim CEOs within different succession patterns. This study observes a recent rise in interim CEO turnover. Prior research suggests that interim CEO succession is fraught with disruption and uncertainty. Despite this, why do interim CEOs continue to successfully navigate these challenges with increasing likelihood? Distinct from other studies, this paper examines the succession patterns of interim CEOs, as well as the influence and mechanisms of their identities and motivations on firm performance. The primary research questions include:

First, how does interim CEO succession influence firm performance?

Second, what is the roles of the interim CEO upon succession, and how do these variations affect firm performance?

Third, through what mediating pathways do interim CEOs impact firm performance?

Further scenario analysis will probe the mediating mechanisms linking interim CEOs and firm performance under diverse succession models.

## Theoretical analysis and research hypotheses

### Interim CEOs and firm performance

According to Ballinger and Marcel (2010) [10], interim succession risks uncertainty. There is a greater likelihood of short-sighted behavior and information asymmetry for interim CEOs during the interim period. Therefore, the performance of firms where the board formally appoints a CEO to succeed them performs better than that of firms with interim appointments. We argue that interim CEO succession has the destructiveness of executive succession and the uncertainty of interim succession. Such dual challenges can have a substantial negative impact on firm performance. This includes potential successors and transition successors.

First, interim CEOs need more legitimacy and authority and face a power vacuum and information asymmetry [15]. The interim CEO’s lack of authority leads to low internal employee cooperation and internal conflict within the executive team. This disadvantage leads to slow strategic decision-making. Secondly, interim succession is a particular type of short-term succession, which makes it challenging to gain the trust of the board of directors and stakeholders in a short period. The limited power will affect firm performance to some extent.

Second, interim CEOs are likelier to have short-sighted behaviors to maintain the firm’s daily operations and have shorter tenures [10]. The short-sighted behavior of executives is either to be promoted to a permanent position [20]. Or they want to make achievements and gain market recognition during the short-acting period. This is seen in the interim CEO’s choice of investments. They often pick options that don’t maximize the firm’s value but offer quick returns. They may also reduce investments that benefit the firm, such as reducing long-term investments [15], reducing R&D investments [11], and so on.

Third, choosing and appointing the CEO is one of the most crucial decisions made by the board of directors. According to attribution theory, individuals analyze their own or others’ behaviors and infer the reasons behind these actions. The manner in which attributions are made influences subsequent behaviors and motivation strength. An interim CEO succession may convey a negative message to stakeholders, implying the firm lacks a plan for executive turnover. This is often seen as a sign of board governance failure [21]. Consequently, it triggers negative sentiments and attributions among investors, external markets, and the media, ultimately impacting firm performance.

Fourth, the appointment of an interim CEO could pose challenges for firms, such as stagnation in strategic transformation and a deficiency in innovation. A result of factors such as the importance the interim CEO placed on reputation and the risk of organisational change. Interim CEOs, as temporary transitioners of the firm, are less likely to turn over or remain in office. Interim CEOs often avoid risk, reluctant to take on responsibility during their tenure. This may lead firms to persist with current strategies, resulting in strategic change stagnation [4]. Thus, slow and less innovative corporate strategic change will lead to lower performance. Based on the above analyses, we propose the hypothesis:

**H1**: Interim CEO succession is negatively related to firm performance.

The aforementioned study concluded that interim CEO succession adversely affects various aspects of business performance. Nevertheless, in recent years, the incidence of interim CEO succession has been on the rise, with numerous instances of interim CEOs taking over. Given this knowledge, why do boards of directors opt for such succession? This paper posits that the rationale behind this decision is closely linked to the circumstances surrounding the former CEO’s departure. For instance, in unforeseen circumstances such as the demise of the former CEO or their involvement in a scandal, the successor might not have been prepared in advance. Such crisis situations create an urgent need for an interim CEO transition. Another scenario involves a former CEO who has served an extended period and gradually become disengaged from the board’s management. The board may seek to recruit an external executive to redefine strategies but fears this could disrupt the existing executive team’s cooperation and unity. In such cases, the board designates the externally recruited executive as an “interim CEO,” while strategically seeking an opportunity to transition “internal candidates” into the new role. This highlights the importance of defining and examining the roles of an interim CEO’s successor.

### Two roles of interim CEOs and firm performance

According to Mooney et al. (2013) [4], interim succession serves as a contingency measure. This measure is bifurcated into long-term and short-term perspectives. Interim CEOs chosen from a long-term perspective are deemed ‘potential successors,’ whereas those selected from a short-term viewpoint are considered ‘transition successors’. A description of the two roles of interim CEO classification is shown in Table 1.

**Table 1:** Interim CEO classification and description.

Interim CEO Category	Succession Planning Perspective	Motivation and Function	Reason for Departure of Predecessor
Potential Interim CEOs	Long-term	Saving the day: strategic change, improving performance	Dismissal, retirement, expiration, etc.
Transition Interim CEOs	Short-term	Stable the day: giving time to find the rightful owner	Separation, death, involvement, etc.

An interim CEO is more likely to be recognized as a ‘potential successor’ when the succession plan is devised from a long-term perspective. In such cases, the departure of the former CEO is often anticipated by the board of directors due to foreseeable reasons such as dismissal or retirement. Potential successors typically emerge from within the company. Research suggests that a predictable CEO transition orchestrated by the board may correlate with diminished organizational performance [16, 22]. Under these circumstances, it becomes strategically crucial for an interim CEO to assume office. The likelihood of turnover for the interim CEO increases, as the company simply uses the interim role as an evaluation period for CEO candidates [23].

Interim CEOs are selected with a short-term focus, often perceived as ‘transition successors.’ Their appointment typically arises from unexpected events such as the abrupt resignation, death, or legal challenges confronting the former CEO. In these scenarios, the board of directors faces constrained time to nominate a new leader. To bridge the period between the sudden exit of the predecessor and the appointment of a permanent CEO, the board usually appoints an interim successor. Given that the selection of an interim CEO is predicated on immediate and uncontrollable circumstances, their role is not to ‘save the day’ but rather to ‘stable the day.’ Drawing from the study by Lian et al. (2020) [14], this paper examines and conceptualizes whether the role of the interim CEO is that of a “potential successor” or a “transition successor.” The classification uses the reasons behind the former CEO’s departure as the defining criterion.

First, potential interim CEOs can effectively alleviate the adverse effects on firm performance due to concerns like a lack of legitimacy and authority, information asymmetry, and executive team turnover. On the one hand, potential successors typically originate from the executive team [6], possessing familiarity with the firm’s routine business and management activities. And they already wield a degree of influence within the firm. On the other hand, potential interim CEO succession helps to enhance the executive team’s stability. Such succession can mitigate the risk of instability and restructuring of the senior management team after the departure of senior executives [24].

Second, the turnaround incentives of potential interim CEOs can have a binding effect during the period, thereby mitigating the negative impact on firm performance. Expectancy theory posits that humans consistently strive to fulfill needs and achieve goals. In contrast to transition interim CEOs, potential interim CEOs anticipate a ‘turnaround’ based on their exemplary performance during the period, thereby shaping their own behavior. As a result, the potential interim CEO is strongly motivated to demonstrate his or her capabilities to the board of directors throughout their tenure. Ultimately, they aim to facilitate a smooth transition for the company or even achieve a ‘turnaround’ to profitability. Therefore, incentives for promotion can effectively offset the adverse effects of inaction, misallocation of resources, and shortsightedness on firm performance during the interim period when potential successors are in place.

Third, potential successors have more substantial incentives to make strategic changes. This motivation can effectively mitigate the negative impact of stagnant strategic changes of the interim CEO on firm performance. On the one hand, based on the psychological commitment view, new executives usually change the rigid management model due to organizational inertia to demonstrate strategic decisions that distinguish them from their predecessors [25]. On the other hand, the departure of a potential successor’s former CEO is commonly due to reasons like dismissal. To some degree, these reasons suggest the board’s intent for strategic transformation [26]. Consequently, potential interim CEOs are more inclined to alter the current state and implement strategic changes to fulfill the board’s mandate for strategic shifts. Based on the above analyses, we propose the hypothesis:

**H2:** Transition interim CEOs correlated more significantly with firm performance in a negative way compared to potential interim CEOs.

### Interim CEO, agency efficiency and firm performance

Agency theory suggests agency problems arise when managers and shareholders have conflicts of interest or information asymmetry. These are among the most critical issues in corporate governance. The first type of agency problem can be divided into higher agency costs and lower agency efficiency [27, 28] Agency costs are more responsive to the costs incurred by self-interested behaviors such as on-the-job spending by executives during their tenure. During their tenure, executives make wrong decisions or fail to increase the company’s business income, which will not only lead to low agency efficiency, but also aggravate agency problems [29].

In our view, the interim CEO has a power vacuum and lacks legitimacy during his or her tenure. These problems can lead to executives not being trusted by the board [15]. Therefore, self-interested behavior, such as on-the-job spending, is less likely to occur. If there is a problem, it is more likely to be of a short-term contractual nature. Specifically, to avoid risks and responsibilities, senior executives did not actively carry out strategic changes, resulting in reduced operating income and other behaviors. And the potential successor of the interim CEOs has the incentive to improve short-term performance and smooth transition. Therefore, they are more motivated to decrease long-term investments, research and development expenditures, and other actions that do not contribute to corporate value during their tenure. Consequently, this paper concludes that there exists an “interim CEO—agency efficiency—firm performance” path between the interim CEO and firm performance. Specifically, the interim CEO’s inaction and evasion of responsibility during the tenure led to the reduction of agency efficiency and exacerbated agency problems. It will negatively affect firm performance. Based on the above analyses, this paper proposes the following hypotheses:

**H3**: Lower agency efficiency negatively mediates the relationship between interim CEO succession and firm performance.

**Fig 1:**
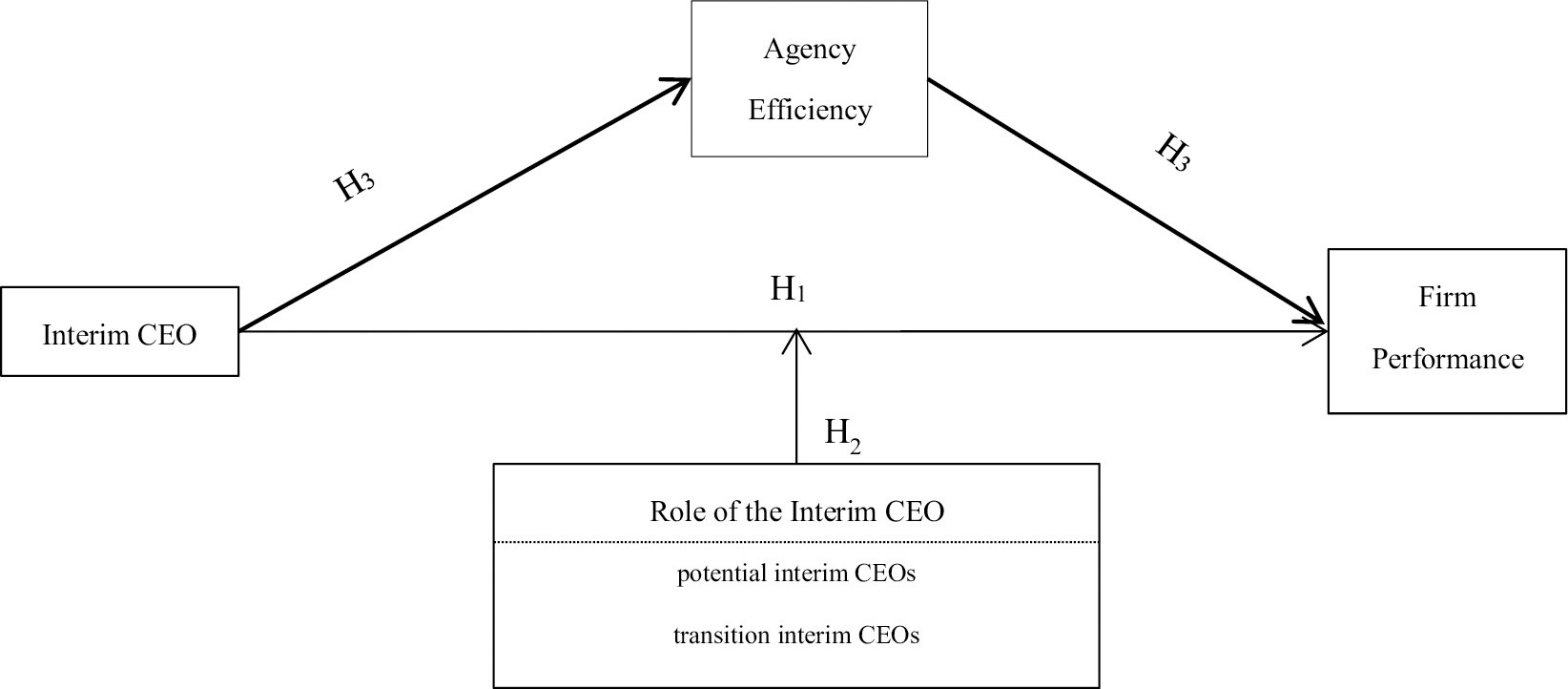
Conceptual framework: Impact of interim CEOs on firm performance.

Fig 1 shows the conceptual framework of the paper (Fig 1).

## Research design

### Sample selection and data sources

This study focuses on A-share listed companies in Shanghai and Shenzhen that experienced interim CEO succession between 2009 and 2019. The study is timed to commence at the end of the 2009 financial crisis, using the economic resurgence as a starting point to explore the dynamics of the subsequent round of economic growth. In 2020, the onset of COVID-19 pneumonia will deliver another substantial blow to the economy. Therefore, selecting the period between the financial crisis and before the COVID-19 outbreak for the study is both comprehensive and rational.

The selection of A-share companies listed in Shanghai and Shenzhen as research subjects is grounded on three justifications. Firstly, A-share markets in Shanghai and Shenzhen provide extensive data. They cover various sectors, including the main board, small and medium-sized boards, and the GEM, representing China’s largest stock market. Secondly, A-share listed companies must follow a stringent information disclosure system. This ensures the openness, transparency, and credibility of their data, guaranteeing its authenticity and reliability. Thirdly, studying these companies is significant to the Chinese economy as the A-share market is considered a barometer of China’s economic health, making the study of A-share listed companies more representative.

In this study, data was filtered based on specific criteria. (1) We omitted listed firms in the finance and insurance sectors. (2) Companies whose corporate nature couldn’t be ascertained were excluded. (3) We disregarded firms under special treatments, such as ST and PT. (4) Listed companies with severely missing data were also eliminated from our study. We obtained 2371 observations from 1443 listed companies. The primary data source is the CSMAR database, including the CEO’s interim situation, the CEO’s essential characteristics, the firm’s basic characteristics, and the firm’s financial data. Considering the sample size of this paper and the effect of extreme values, the leading continuous variables are shrink-tailed by 1% and 99%. In addition, our study controls for year dummy variables (Year) and industry dummy variables (Industry) in all regression analyses.

### Variable definition and model setting

#### 1. Explained variables

We select return on assets (ROA) as the explanatory variable. Given that this paper explores issues related to interim CEO succession, more consideration is given to the impact on short-term corporate performance. Furthermore, the return on assets, employed as a metric to gauge corporate performance, exhibits enhanced comparability and stability, rendering it more apt for assessing short-term enterprise performance.

#### 2. Explanatory variables

Interim CEO (Ifagent) mainly refers to the change of general manager, i.e., interim general manager. It is measured according to whether the successor is an interim successor when the general manager of the enterprise changes in the year. If the successor is serving in an interim capacity, a value of 1 is assigned; otherwise, a value of 0 is given. The data are mainly obtained from the CSMAR database and manually collated. In some enterprises, consecutive changes of the same general manager in one year exist. Observing duplicate instances of the same general manager undergoing changes within a single year can confound empirical outcomes. Consequently, the abbreviated tenure of the interim representative is unlikely to exert a significant influence on the organization. This paper removes observations where the interim tenure is at most three months for consecutive changes of the same successor within a year. The purpose of treating consecutive changes is to examine the interim CEO quickly, after which there is a greater likelihood of a formal succession.

#### 3. Moderating variables

This study extends the work of Mooney et al. (2013) [4] and Lian et al. (2020) [14], which categorized the succession status of interim CEOs into potential successors and transition successors, contingent on the predecessor CEO’s departure reasons. Among them, when the former CEO leaves the company due to job transfer, retirement, expiration of term, change of controlling interest, termination of employment, improvement of corporate structure, and other reasons, the interim CEO is regarded as a potential successor. When the former CEO resigns, health reasons, personal reasons, involvement in a case, and the end of the agency, the interim CEO is regarded as a transition successor.

#### 4. Mediating variables

Drawing on the study of Liu and Gao (2015) [29], the mediating variable, agency efficiency (Efficiency) is measured by the total asset turnover ratio. Agency efficiency is calculated by the following formula: operating income/total assets ending balance.

#### 5. Control variables

In order to control the impact of other factors on corporate performance, we draw on the studies of Lian et al. (2020) [14] and Yun and Ning (2020) [30], and control for some variables. The specific definitions of each variable are shown in Table 2.

**Table 2:** Variable definitions and descriptions.

Variable Type	Variable Name	Variable Code	Variable Definition
Dependent Variable	Firm Performance	ROA	Return on assets
Independent Variable	Interim CEO	Ifagent	The value is 1 if the general manager is interim successor, and 0 if not
Moderator Variable	Role of the Interim CEO	Role	Potential interim CEOs are assigned a value of 1, and transition interim CEOs are assigned a value of 0
Mediator Variable	Agency Efficiency	Efficiency	Total asset turnover ratio
Control Variables	Enterprise Lifetime	Time	Establishment years of enterprises
	Enterprise Size	Size	Natural logarithm of total assets of the enterprise at the end of the period
	Board Size	Boardsize	Number of directors
	Executive Shareholding Ratio	Gshare	Number of shares held by executives/total shares
	Ownership Concentration	Top1	Share proportion of the largest shareholder
	Share Structure	Change	No change in the shareholding structure is assigned a value of 1, and a change is assigned a value of 0
	Management Expense Ratio	MFR	Administrative expenses/operating income
	Volatility of Earnings	ProfitsV	(EBIT/total assets) three-year volatility; Where: Three-year volatility calculated: t − 2 to t year standard deviation
	Proportion of Tangible Assets	Tang	Net fixed assets/total assets
	Asset-Liability Ratio	Lev	Total liabilities/total assets
	CEO Duality	Dual	Whether the chairman of the company concurrently serves as the general manager, the value assigned to the concurrent position is 1, otherwise it is 0
	Year of Belonging	Year	Year dummy variable
	Industry of Belonging	Industry	Industry dummy variable

#### 6. Model setting

Eqs (1)–(3) are constructed to verify the effects of interim CEO and firm performance and its mediating mechanism:

$${ROA}_{i,t}=\alpha_{0}+\alpha_{1}{Ifagent}_{i,t}+\alpha_{i}{Controls}_{i,t}+\Sigma Industry+\Sigma Year+\varepsilon_{i,t}$$ (1)

$${Efficiency}_{i,t}=\alpha_{0}+\alpha_{1}{Ifagent}_{i,t}+\alpha_{i}{Controls}_{i,t}+\Sigma Industry+\Sigma Year+\varepsilon_{i,t}$$ (2)

$${ROA}_{i,t}=\alpha_{0}+\alpha_{1}{Ifagent}_{i,t}+\alpha_{2}{Efficiency}_{i,t}+\alpha_{i}{Controls}_{i,t}+\Sigma Industry+\Sigma Year+\varepsilon_{i,t}$$ (3)

The impact of two roles of interim CEOs on firm performance is regressed by dividing the sample into two groups according to whether the interim CEO is a potential or a transition successor. The moderating effect of different succession roles of interim CEOs on the relationship between interim succession and firm performance is judged according to the coefficients and significance. Where *i* denotes firm, *t* denotes year, and *Controls* denotes all control variables. Regression analyses control for both year and industry-fixed effects and are Cluster-adjusted for standard errors at the firm level.

## Empirical results and analyses

### Descriptive statistics analysis

The descriptive statistics of the main variables in this paper are shown in Table 3. The results show that the mean value of interim CEO succession is 0.067, with a standard deviation of 0.249. The mean value of firm performance is 0.029, with a standard deviation of 0.067. The sample enterprises in which interim CEO succession occurs accounted for 6.7% of the sample enterprises. The moderator variable Role has a mean of 0.677 and a standard deviation of 0.468. The results indicate that there are more potential interim CEOs than transitional interim CEOs in the sample of interim succession.

**Table 3:** The mean, median, standard deviation, maximum and minimum.

Variable	Mean	Median	Standard Deviation	Maximum Value	Minimum Value
ROA	0. 029	0. 030	0. 067	0. 185	-0. 340
Ifagent	0. 067	0.000	0. 249	1.000	0.000
Role	0. 677	1.000	0. 468	1.000	0.000
Time	16. 790	17.000	5. 651	31.000	4.000
Size	22. 270	22. 070	1. 295	26. 500	19. 850
Boardsize	8. 655	9.000	1. 718	15.000	5.000
Gshare	0. 049	0.000	0. 111	0. 554	0.000
Top1	34. 250	32. 020	14. 910	74. 820	8. 500
Change	0. 233	0.000	0. 423	1.000	0.000
MFR	0. 095	0. 076	0. 079	0. 496	0. 007
ProfitsV	0. 034	0. 018	0. 048	0. 311	0. 001
Tang	0. 221	0. 185	0. 167	0. 714	0. 003
Lev	0. 451	0. 447	0. 202	0. 907	0. 065
Dual	0. 166	0.000	0. 372	1.000	0.000

### Correlation analysis

The correlation analysis between the variables shows that the coefficient between the interim CEO (Ifagent) and the firm performance (ROA) is -0.042 and is significant at the 5% level. The correlation analysis results preliminarily verified hypothesis H1. The correlation coefficients between the variables in the model are all less than 0.5. The Variance Inflation Factor (VIF) of the regression equations is less than 5, which suggests that there is no severe problem of multi-collinearity.

### Multiple regression analysis

Table 4 reports the group test results of interim CEOs, firm performance, and different succession roles. Column (1) interim CEO (Ifagent) is significantly negatively correlated with firm performance (ROA) at the 5% level (coef. = -0.013, p<0.05). The test results support hypothesis H1. Columns (2) and (3) are the results of the grouped regression tests of transitional interim CEOs and potential interim CEOs. Column (2) interim CEO (Ifagent) is significantly and negatively related to firm performance (ROA) at the 10% level (coef. = -0.013, p<0.1). The coefficient of interim CEO and firm performance in column (3) is -0.010 and the result is not significant. The subgroup regression results indicate that the succession of potential interim CEOs that firms have in reserve has a less negative impact on firm performance than transitional interim CEOs. Hypothesis H2 is verified.

**Table 4:** Regression results of interim CEO and firm performance.

	Full sample	Transitional CEO sample	Potential CEO sample
	(1)	(2)	(3)
	ROA	ROA	ROA
Ifagent	-0. 013**	-0. 013*	-0. 010
	(-2. 409)	(-1. 857)	(-1. 615)
Time	0. 001***	0. 002***	0. 001**
	(3. 381)	(3. 593)	(2. 007)
Size	0. 004***	0. 005**	0. 005***
	(3. 260)	(2. 113)	(3. 179)
Boardsize	0. 001	-0. 001	0. 002**
	(1. 538)	(-0. 542)	(2. 054)
Gshare	0. 017	0. 008	0. 024
	(1. 332)	(0. 423)	(1. 450)
Top1	0. 000***	0. 000***	0. 000***
	(4. 181)	(2. 613)	(3. 378)
Change	-0. 003	0. 005	-0. 006*
	(-1. 151)	(0. 843)	(-1. 860)
MFR	-0. 183***	-0. 180***	-0. 190***
	(-7. 372)	(-4. 704)	(-5. 946)
ProfitsV	-0. 472***	-0. 506***	-0. 429***
	(-6. 389)	(-4. 150)	(-4. 871)
Tang	-0. 044***	-0. 057***	-0. 045***
	(-4. 192)	(-2. 992)	(-3. 657)
Lev	-0. 121***	-0. 094***	-0. 135***
	(-12. 124)	(-4. 749)	(-12. 767)
Dual	0. 002	0. 003	0. 002
	(0. 437)	(0. 435)	(0. 428)
Constant	0. 004	0. 004	-0. 012
	(0. 111)	(0. 073)	(-0. 283)
Year/Industry	Yes	Yes	Yes
N	2 371	767	1 604
Adj_R2	0. 347	0. 357	0. 349

Note

***p<0.01

**p<0.05

*p<0.1, t-values in parentheses, standard errors for all tests are clustered at the firm level and adjusted for heteroskedasticity, below.

Table 5 presents the test results of the mediating mechanism between the interim CEO and firm performance. Column (2) the coefficient of interim CEO (Ifagent) is significantly and negatively related to agency efficiency at the 10% level (coef. = -0.047, p<0.1). The coefficient of column (3) agent efficiency is significantly and positively correlated with firm performance (ROA) at the 1% level (coef. = 0.014, p<0.01). The coefficient of Ifagent is negatively significant at the 5% level. Agent efficiency passed the mediation mechanism test and played a partial mediating role. This indicates that interim CEOs lead to lower firm performance due to lower agency efficiency during their tenure, and hypothesis H3 is tested.

**Table 5:** Regression results of the mediating mechanism between interim CEO and firm performance.

	(1)	(2)	(3)
	ROA	Efficiency	ROA
Ifagent	-0. 013**	-0. 047*	-0. 012**
	(-2. 409)	(-1. 814)	(-2. 306)
Efficiency			0. 014***
			(3. 525)
Time	0. 001***	0. 001	0. 001***
	(3. 381)	(0. 276)	(3. 340)
Size	0. 004***	-0. 050***	0. 005***
	(3. 260)	(-4. 288)	(3. 775)
Boardsize	0. 001	0. 012*	0. 001
	(1. 538)	(1. 795)	(1. 318)
Gshare	0. 017	-0. 059	0. 018
	(1. 332)	(-0. 781)	(1. 411)
Top1	0. 000***	0. 003***	0. 000***
	(4. 181)	(3. 626)	(3. 711)
Change	-0. 003	0. 023	-0. 004
	(-1. 151)	(1. 058)	(-1. 267)
MFR	-0. 183***	-2. 313***	-0. 151***
	(-7. 372)	(-14. 866)	(-5. 634)
ProfitsV	-0. 472***	0. 488**	-0. 479***
	(-6. 389)	(2. 253)	(-6. 439)
Tang	-0. 044***	-0. 156**	-0. 042***
	(-4. 192)	(-1. 984)	(-3. 932)
Lev	-0. 121***	0. 295***	-0. 125***
	(-12. 124)	(4. 532)	(-12. 589)
Dual	0. 002	-0. 011	0. 002
	(0. 437)	(-0. 527)	(0. 480)
Constant	0. 004	1. 502***	-0. 017
	(0. 111)	(5. 793)	(-0. 471)
Year/Industry	Yes	Yes	Yes
N	2 371	2 371	2 371
Adj_R2	0. 347	0. 423	0. 352

## Robustness test

### Endogeneity test: Propensity score matching method (PSM)

Considering that the interim CEO succession event may not be completely exogenous, there may be endogeneity problems caused by sample selectivity bias. In this paper, concerning the study of Lian et al. (2020) [14], the propensity score matching method (PSM) is used to reduce the regression bias due to sample selection bias. Drawing upon existing literature, this study selects enterprise lifetime, enterprise size, executive shareholding ratio, proportion of tangible assets, ownership concentration, asset-liability ratio, and volatility of earnings as matching variables. These are chosen based on the control variables previously discussed in this paper. Moreover, the above matching and independent variables (Ifagent) were subjected to 1:3 pairing and Logit regression. The results of the test show that the balanced performance test is favorable. The regression was conducted again using the matched data. As shown in column (2) of Table 6, the coefficient between interim CEO (Ifagent) and firm performance (ROA) is -0.018 and significant at a 1% level. The empirical results show that the central hypothesis of this paper is robust.

**Table 6:** Test results of propensity score matching and Heckman two-stage method.

	(1) Before matching	(2) After matching	(3) Heckman second stage
	ROA	ROA	ROA
Ifagent	-0. 013**	-0. 018***	-0. 013**
	(-2. 409)	(-2. 982)	(-2. 496)
Imr			-0. 070***
			(-3. 718)
Constant	0. 004	0. 000	0. 144***
	(0. 111)	(0. 004)	(2. 850)
Controls	Yes	Yes	Yes
Year/Industry	Yes	Yes	Yes
N	2 371	590	2 371
Adj_R^2^	0. 347	0. 361	0. 353

Note: Control variables have been omitted, as below.

### Endogeneity test: Heckman two-stage

Considering that samples in which interim succession occurs may also suffer from self-selection estimation bias. We further use the Heckman two-stage method for endogeneity testing. A Probit analytical model regarding firm performance (ROAdummy) is developed. Specifically, in the first stage, refer to Zhu et al. (2021) study [31]. This paper’s instrumental and main control variables are selected as explanatory variables. The instrumental variables are selected concerning Lian et al. (2020) study [14]. We select the total number of interim CEOs in the region (City) and the total number of interim CEOs in the province as the instrumental variables. The inverse Mills ratio calculated in the first stage is then put into the second stage equation for regression. The regression results are shown in column (3) of Table 6, where the inverse Mills ratio (Imr) and the explanatory variables (Ifagent) are significant. The test results remain consistent with the previous results.

### Other robustness tests

#### 1. Replacement of variables

This paper refers to the research of Yang et al. (2021) [32] scholars, replacing the dependent variable with the return on equity (ROE) of the enterprise. It can also reflect the short-term performance of the enterprise. The replaced and independent variables are re-examined empirically, and the test results are shown in Table 7. The coefficient of interim CEO (Ifagent) in column (1) is negatively significant at the 10% level, and hypothesis H1 is tested again. Subgroup regression results show that the coefficient of interim CEO (Ifagent) in column (4) is negatively significant at the 10% level for the transitional CEO group. The potential CEO group’s coefficient is insignificant. The test results support hypothesis H2. The mediation test results show that the coefficient of interim CEO (Ifagent) in column (2) is significantly negatively correlated with agency efficiency at the 10% level. And the coefficient of agency efficiency in column (3) is significantly negatively correlated with agency efficiency at the 10% level. The coefficient of column (3) Efficiency is significantly and positively related to firm performance (ROE) at the 5% level, and hypothesis H3 is tested.

**Table 7:** Regression results after replacing variables.

		Full sample		Transitional CEO sample	Potential CEO sample
	(1)	(2)	(3)	(4)	(5)
	ROE	Efficiency	ROE	ROE	ROE
Ifagent	-0. 023*	-0. 047*	-0. 022*	-0. 028*	-0. 016
	(-1. 954)	(-1. 814)	(-1. 884)	(-1. 748)	(-1. 177)
Efficiency			0. 020**		
			(1. 991)		
Constant	-0. 126*	1. 502***	-0. 156**	-0. 035	-0. 172*
	(-1. 725)	(5. 793)	(-2. 138)	(-0. 307)	(-1. 883)
Controls	Yes	Yes	Yes	Yes	Yes
Year/Industry	Yes	Yes	Yes	Yes	Yes
N	2 371	2 371	2 371	767	1 604
Adj_R^2^	0. 252	0. 423	0. 254	0. 298	0. 240

#### 2. Replacement of interim succession samples

The chairman of the board and the general manager are equally crucial to the firm and have a decisive impact on the firm’s business decisions. We replace the sample of succession of interim general manager with the sample of succession of interim chairman. Table 8 shows the test results of interim succession and firm performance, the heterogeneity of succession identity, and the intermediary mechanism. The empirical results support the hypotheses H1 and H3, and the hypothesis H2 has not been verified for the time being.

**Table 8:** Regression results after replacing samples.

		Full sample		Transitional CEO sample	Potential CEO sample
	(1)	(2)	(3)	(4)	(5)
	ROA	Efficiency	ROA	ROA	ROA
Ifagent	-0. 011**	0. 003	-0. 011**	-0. 012***	-0. 003
	(-2. 408)	(0. 114)	(-2. 429)	(-2. 785)	(-0. 265)
Efficiency			0. 010*		
			(1. 771)		
Constant	-0. 035	1. 469***	-0. 049	-0. 034	0. 013
	(-0. 769)	(4. 443)	(-1. 087)	(-0. 710)	(0. 102)
Controls	Yes	Yes	Yes	Yes	Yes
Year/Industry	Yes	Yes	Yes	Yes	Yes
N	1 287	1 287	1 287	1 043	244
Adj_R^2^	0. 387	0. 442	0. 389	0. 373	0. 478

Note: The interim chairman’s sample has 1,287 valid observations due to the replacement of the sample.

## Mechanism analysis

This paper’s research has found that interim CEOs can be classified into two roles: potential successors and transition successors. While it is true that the succession of an interim CEO has a negative impact on firm performance in the short term, potential successors have less negative impact than transition successors. Therefore, the focus of this paper is to investigate what kind of interim CEOs can reduce the negative impact on firm performance. Would more effective incentives for a potential successor CEO who wants to make a successful transition help them to “save the day”? For the transition successor, can the board of directors assist the interim CEO in “stable the day” by selecting a competent person and implementing effective monitoring measures?

**Fig 2:**
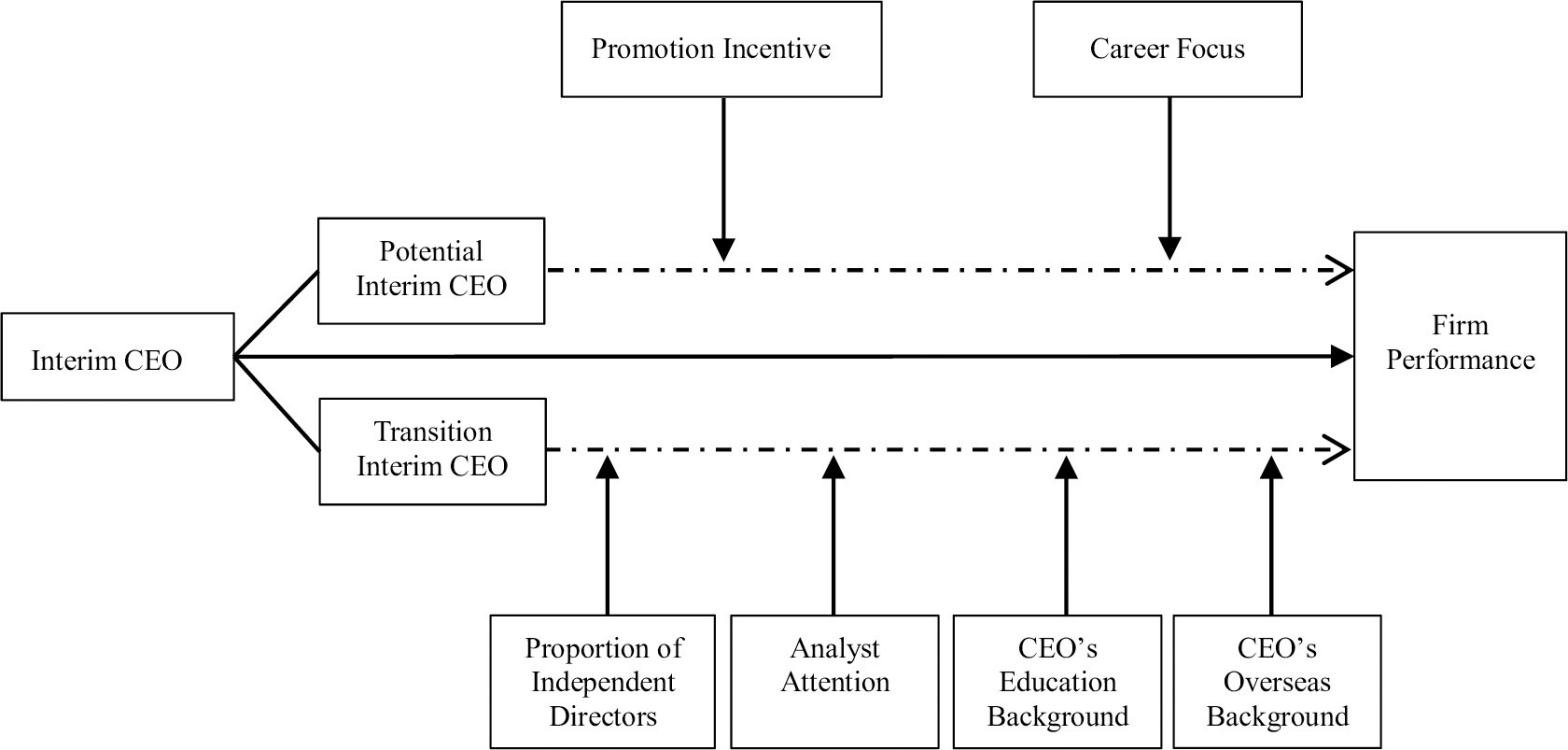
Mechanism analysis: Two roles of interim CEOs on firm performance.

Effective oversight measures and the appointment of competent interim CEOs can alleviate the adverse effects of the transition successor on company performance. This study characterizes the tenure of potential interim CEOs as a ‘transition period.’ During this period, effective incentives for potential successors can better help firms to make a smooth transition. We aim to further investigate: firstly, the direct moderating mechanism of executive incentives (promotion incentive, career focus) on the performance of potential successor and firms. Secondly, the moderating mechanisms of executive ability (education background, overseas background) and monitoring constraints (independent director ratio, analyst attention) on the transition interim CEO and firm performance. The details are shown in Fig 2.

### Moderating mechanisms of potential interim CEOs and firm performance

#### 1. The moderating role of promotion incentive

Executive incentives encompass behaviors that elicit appropriate motivation, incorporating both material and non-material rewards, such as advancement in position accompanied by a corresponding salary increase. The “tournament theory” proposed by Lazear and Rosen (1981) [33] suggests that the purpose of incentives can be made more effective by differentiating the pay gap between different management levels.

For potential interim CEOs, promotion incentives can help firms mitigate the negative impact of turnover events and temporary succession on firm performance. Compared to transitional interim successors, potential interim CEOs have a higher probability of promotion. Contemplating future career trajectories can alleviate the prolonged impact on company performance resulting from myopic actions. Secondly, advancement can bolster the interim CEO’s reputation within the firm and reinforce their standing on the board of directors or the executive team. This, in turn, can partially alleviate the interim successor’s deficiency in legitimacy and authority. Finally, potential interim CEOs have some degree of incentive to promote, which can improve their risk tolerance and thus implement more risky investments [34].

We further conduct a grouped regression of promotion incentives of potential interim CEOs. The regression results are shown in columns (1) and (2) of Table 9. The coefficient of interim CEOs (Ifagent) in column (1) is -0.015, which is significant at the 10% level. The coefficient of interim CEOs (Ifagent) in column (2) is 0.003. The grouping results indicate that the negative impact of interim succession on firm performance is significantly mitigated when potentially succeeding interim CEOs have high promotion incentives.

**Table 9:** Moderating mechanism of potential interim CEOs and firm performance: Executive incentives.

	ROA			
	(1)	(2)	(3)	(4)
	Low promotion incentive	High promotion incentive	Low career focus	High career focus
Ifagent	-0. 015*	0. 003	-0. 013**	0. 007
	(-1. 761)	(0. 343)	(-2. 204)	(0. 315)
Constant	0. 014	0. 038	0. 050	-0. 091
	(0. 248)	(0. 914)	(0. 885)	(-1. 641)
Controls	Yes	Yes	Yes	Yes
Year/Industry	Yes	Yes	Yes	Yes
N	796	808	847	757
Adj_R^2^	0. 380	0. 302	0. 380	0. 298

#### 2. The moderating role of career focus

CEO reputation is a rating given by the market and can directly affect the future career development of executives. For prospective interim CEOs with internal backups, career orientation serves as a tacit incentive that can spur managers to diligently cultivate a positive reputation and facilitate an expedited turnover. In addition, the level of career focus of executives directly affects their own diligence and talent development [35].

On the one hand, internal and external stakeholders concern the way of interim succession. Therefore, helping the company through the turbulent environment can better enhance the reputation of the potential interim CEO in the manager market. In addition, CEOs with high career focus are likely to have yet to establish their reputation and urgently need to make achievements during their tenure [36] to enhance the probability of turnover. On the other hand, CEOs with a high career focus are better able to discipline their behavior for the sake of their reputation and future careers [37]. Thus, potential interim CEOs’ career concerns can mitigate the negative impact on firm performance to a certain extent.

Career focus in this paper draws on the study of Yun and Ning (2020), and group regression is conducted based on the mean of CEO age. The test results are shown in columns (3) and (4) of Table 9. The coefficient of the lower career focus group is -0.013, which is significant at the 5% level, and the coefficient of the higher career focus group is 0.007. The grouped regression results support the theoretical logic of this paper.

### Moderating mechanisms of transition interim CEOs and firm performance

#### 1. The moderating role of executive ability

Upper echelons theory posits that attributes such as managers’ educational background, personal history, age, and gender distinctly influence company performance. Kaplan et al. (2012) [38] found that firm performance is related to CEO competence. In this paper, transition interim CEOs’ education and overseas background can better reflect their individual capabilities.

Initially, transition interim CEOs possessing advanced academic qualifications or international experience can devise more specialized, holistic, and innovative solutions when confronted with corporate adversity [39]. This can offer corporations novel viewpoints and methodologies, enhancing the likelihood that the executive team will acknowledge and esteem their exceptional personal aptitudes. Second, transition interim CEOs with overseas backgrounds are more concerned with the effectiveness of governance and the maintenance of their reputation [40]. This weakens, to some extent, short-sighted behaviors such as risky investment reductions arising from the interim succession. Finally, capable transition interim CEOs are also more risk-tolerant and willing to make changes to improve firm performance [41].

Based on this, the regressions were conducted by dividing the transition interim CEOs into two groups of high and low in terms of their education and overseas background. Executives with a bachelor’s degree or higher are considered to be relatively well-educated. The test results are shown in Table 10. The results in columns (1) and (2) show that the coefficient of the lower academic qualification group is -0.032 and is significant at the 10% level compared to the transition interim succession group with higher group. Columns (3) and (4) reveal that the non-overseas group’s coefficient is -0.018, significant at the 5% level compared to its overseas-experienced counterpart. The test results indicate that the negative impact on firm performance can be weakened when the transition interim CEO has a high academic qualification or overseas background.

**Table 10:** Moderating mechanism of transition interim CEOs and firm performance: Executive ability.

	(1)	(2)	(3)	(4)
	Low academic qualification	High academic qualification	No overseas background	Have overseas background
	ROA	ROA	ROA	ROA
Ifagent	-0. 032*	-0. 007	-0. 018**	0. 014
	(-1. 792)	(-0. 577)	(-2. 261)	(0. 264)
Constant	-0. 024	-0. 139	-0. 021	0. 184
	(-0. 202)	(-1. 502)	(-0. 265)	(0. 515)
Controls	Yes	Yes	Yes	Yes
Year/Industry	Yes	Yes	Yes	Yes
N	340	427	696	71
Adj_R^2^	0. 239	0. 458	0. 403	0. 184

#### 2. The moderating role of internal and external monitoring

Based on the perspective of individual heterogeneity of interim CEOs, the above analyses investigate how the individual capabilities of transitioning interim CEOs mitigate the negative impact of temporary succession events on firm performance. We will further analyze whether the internal and external supervision mechanism can play a better restraint role during the transition interim CEO term.

Pertaining to internal and external oversight, the independent director framework, as a mechanism addressing agency issues, can curb the opportunistic actions of controlling shareholders and managers [42]. Transition interim CEOs are prone to short-sighted behaviors, wrong investments, and other agency problems due to their short-term contractual nature. Moreover, by effectively linking internal and external stakeholders, independent directors can mitigate the agency conflict problem between executives and shareholders. In terms of external supervision, analysts are effective market intermediaries. By feeding effective information back to the market, analysts can alleviate the problem of information asymmetry [43]. As a result, analysts are concerned that the negative attribution of interim succession events and the negative impact on the business can be mitigated.

Table 11 shows the results of the monitoring mechanism grouping test of independent directors’ and analysts’ attentions. Columns (1) and (2) reveal that interim CEOs’ negative impact on firm performance is more pronounced in groups with fewer independent directors (coef. = -0.034, p<0.1). Columns (3) and (4) indicate that interim CEOs’ adverse impact on performance is heightened in groups with less analyst focus (coef. = -0.034, p<0.05). Thus, empirical tests show independent directors and analysts’ focus enhance regulatory effectiveness, reducing interim succession transitions’ adverse impacts on company outcomes.

**Table 11:** The moderating mechanism of transition interim CEOs and firm performance: Internal and external monitoring.

	(1)	(2)	(3)	(4)
	Low proportion of independent directors	High proportion of independent directors	Low analyst attention	High analyst attention
	ROA	ROA	ROA	ROA
Ifagent	-0. 034*	-0. 006	-0. 034**	0. 005
	(-1. 870)	(-0. 845)	(-2. 361)	(0. 439)
Constant	-0. 083	-0. 086	-0. 087	0. 185*
	(-0. 662)	(-1. 027)	(-0. 840)	(1. 851)
Controls	Yes	Yes	Yes	Yes
Year/Industry	Yes	Yes	Yes	Yes
N	379	388	344	185
Adj_R^2^	0. 394	0. 380	0. 228	0. 608

## Results and discussion

The present study addresses the practical issue of why boards of directors appoint interim executives, despite awareness that interim CEOs can influence firm performance. Previous research has predominantly highlighted the adverse effects of interim CEO succession on firm performance, encompassing negative impacts on firm performance, research and development (R&D), and surplus management [1, 19]. This study demonstrates that interim succession indeed exerts a negative influence on corporate performance, albeit to a certain extent and for a specific duration. However, by considering the high rate of interim CEOs who successfully transition into permanent roles or even lead turnarounds, the findings presented herein diverge significantly from prior studies.

Firstly, this paper adopts the perspective that interim CEOs assume office in different capacities and reveals that not all interim CEOs negatively impact firm performance. Interim CEOs with potential for long-term succession exhibit less detrimental effects on firm performance compared to those serving as temporary placeholders. This study endeavors to bridge the gap between academic research and corporate realities, offering an academic interpretation of a pragmatic status quo.

Second, while interim succession remains a viable strategy for boards of directors, limited research offers tangible solutions for companies facing this challenge. In addressing this gap, the present study delves into moderating mechanisms that can mitigate the negative impact of interim CEO succession on firm performance by taking into account the specific circumstances of firms. This investigation contributes to a more seamless transition for companies in crisis situations and promotes a more effective utilization of interim succession.

## Conclusion

In this study, we categorize interim CEOs into two roles:“potential successors” and “transition successors.” We then investigate the varied impacts that these succeeding interim CEOs have on firm performance under different circumstances. The empirical analysis reveals that:

Interim CEOs who are potential successors have a less detrimental effect on firm performance when compared to transition successors.

The examination of mediating mechanisms suggests that the presence of interim CEOs results in diminished firm performance due to reduced agency efficiency.

Further analysis of moderating mechanisms shows that promotion incentives and the career focus of executives positively influence potential interim CEOs, effectively alleviating the negative impact on firm performance.

Interim CEOs with higher education or overseas background are better positioned to enhance firm performance; moreover, the oversight provided by internal independent directors and the scrutiny of external analysts can exert a restraining influence on transition interim CEOs, thereby mitigating the adverse effects on firm performance.

### Practical insights

Firms should effectively distinguish between “potential successors” and “transition successors” and formulate replacement plans and defense measures. For enterprises that choose potential interim CEOs to succeed, they can formulate detailed executive turnover plans and inspection mechanisms in advance. For transition interim CEOs to succeed, enterprises should find suitable replacements as soon as possible and implement measures to defend against disruptive turnover events, to reduce the risk of uncertainty faced by enterprises.

Enterprises should fully stimulate the role of a smooth transition of interim CEOs and improve the agency efficiency of interim CEOs. Firms should pay attention to the incentive role of potential interim CEOs to prevent them from engaging in short-sighted behaviors such as surplus management for an early turnover. Firms should be targeted when selecting interim successors, matching the personal characteristics and capabilities of transition interim CEOs, among other things, with the firm.

Enterprises and the market should pay attention to the monitoring mechanism for executives by internal and external stakeholders such as independent directors and analysts’ concerns. Through adequate supervision to reduce their avoidance of responsibility and inaction, give full play to the role of discipline and management of managers, and then more effective relief for the enterprise. Give full play to the positive effects of internal governance elements such as reducing uncertainty risk, mitigating agency conflicts, and weakening the short-sighted behavior of executives.

### Limitations

The study in this paper is confined to the duration from the financial crisis’s conclusion to the new crown epidemic’s onset, necessitating an update to the data. We recognize that there may be unobserved limitations despite our best efforts to select appropriate control variables. The sample comes only from A-share firms on China’s Shanghai and Shenzhen bourses, suggesting further study is needed for other nations and SMEs. Moreover, this paper’s categorization of interim CEO roles is solely based on the reasons for the former CEO’s departure, which is somewhat simplistic.

### Future research directions

The criteria distinguishing between transition interim CEOs and potential interim CEOs warrants a more nuanced definition.

Prospective studies could delve deeper into assessing whether potential successors genuinely possess the capacity to enact a turnaround in the near term.

Scholars should focus on how this interim succession method affects various company aspects, including risk-taking propensity and dedication to corporate social responsibility.

## Supporting information

S1 File Original data-interim CEO. (XLSX)
