# Correction: “Timely help” or “one disaster after another”: The impact of potential and transition interim CEO succession on corporate performance

**DOI:** 10.1371/journal.pone.0321325

**Published:** 2025-03-27

**Authors:** Conghui Zhao, Xin Guan, Xinyue Wu

The authors Conghui Zhao, Xin Guan and Xinyue Wu should not have been attributed equal contribution to this work.

## References

[1] Zhao C, Guan X, Wu X. “Timely help” or “one disaster after another”: The impact of potential and transition interim CEO succession on corporate performance. PLoS One. 2024;19(10): e0311281. doi: 10.1371/journal.pone.0311281 39401265 PMC11472958

